# Nivolumab-associated hypopituitarism in a 61-year-old woman with metastatic triple-negative breast

**DOI:** 10.1093/omcr/omag060

**Published:** 2026-05-10

**Authors:** Ketevan Lomidze, Marine Gordeladze, Nino Kikodze, Nino Charkviani, Tinatin Chikovani

**Affiliations:** Department of Immunology, Department of Endocrinology, Tbilisi State Medical University, Vazha-Pshavela Str. #33, Tbilisi 0186, Georgia; Department of Endocrinology, Tbilisi State Medical University, Vazha-Pshavela Str. #33, Tbilisi 0186, Georgia; Department of Immunology, Tbilisi State Medical University, Vazha-Pshavela Str. #33, Tbilisi 0186, Georgia; Department of Endocrinology, Tbilisi State Medical University, Vazha-Pshavela Str. #33, Tbilisi 0186, Georgia; Department of Immunology, Tbilisi State Medical University, Vazha-Pshavela Str. #33, Tbilisi 0186, Georgia

**Keywords:** nivolumab, Hypophysitis, adrenal insufficiency, breast neoplasms, case report

## Abstract

Immune checkpoint inhibitors (ICIs) have transformed the treatment landscape of cancer, enhancing response rates and survival outcomes. However, immune-related adverse events (irAEs), which can affect any organ or system, can limit their use. We report a rare case of hypophysitis in a triple-negative breast cancer patient receiving nivolumab, an anti-PD-1 monoclonal antibody. After six infusions of nivolumab, the patient developed hypocortisolism and hypothyroidism symptoms, such as mood swings, fatigue, unintentional weight loss, and vomiting. The diagnosis was confirmed by laboratory testing. The patient was successfully managed with hormone replacement therapy. Endocrinopathies can be life-threatening, necessitating treating doctors recognise their clinical symptoms, diagnose them, and manage them to ensure effective treatment and enhance the outcomes of immune checkpoint inhibitor-induced endocrine adverse effects.

## Introduction

The emergence of immune checkpoint inhibitors (ICIs) anti-CTLA-4 and anti-PD-1 has revolutionized the treatment of oncological diseases by increasing the response and survival rate of tumours [1]. However, ICI resistance restricts the number of individuals who respond, and immune-related side effects complicate treatment. Immunotherapy can affect any organ or system, and the side effects are usually mild, curable, and reversible; some might be life-threatening and cause permanent illness. The median clinical onset of adverse events for nivolumab is 40 weeks [2].

Among ICI-related adverse events, the third most common are endocrine disorders [1]. Manifestations of adverse endocrine events can include thyroid dysfunction, pituitary inflammation, insulin deficiency, adrenal insufficiency, and, rarely, hypoparathyroidism and arginine vasopressin deficiency.

We present a rare case of nivolumab-induced hypophysitis in a triple-negative breast cancer patient. The patient provided written informed consent for the publication of this case report.

## Case presentation

A 61-year-old female was diagnosed with triple-negative breast cancer with metastatic lesions in the liver. Her oncologist recommended a treatment regimen combining pembrolizumab (anti-PD-1) (trade name Keytruda) and paclitaxel. But the patient, unable to afford the price of Keytruda treatment, received an offer to switch from pembrolizumab to nivolumab (anti-PD-1) under the trade name Opdivo. She tolerated the treatment well, but after the sixth infusion, she began having mood alterations, exhaustion, unintended weight loss (−6 kg), and vomiting. Her oncologist described her as a very optimistic and cheerful person in the past, but now she was apathetic. A previous endocrine disorder was not known. The patient was neither smoking nor consuming alcohol. The patient did not report experiencing severe headaches, diarrhoea, or abdominal pain.

During the checkup, her blood pressure was low (systolic blood pressure, SBP: 96 mmHg, diastolic blood pressure, DBP: 73 mmHg), and her heart rate was 74. Her laboratory assays revealed noteworthy hyponatraemia—128.6 mmol/l (N: 136–146). We examined her pre-treatment laboratory assays. TSH, FT4, and cortisol tests, along with complete blood count tests and other biochemistry assays, are standard procedures for patients receiving immune checkpoint inhibitors. All measurements were either normal or showed no clinically significant changes.

**Figure 1:**
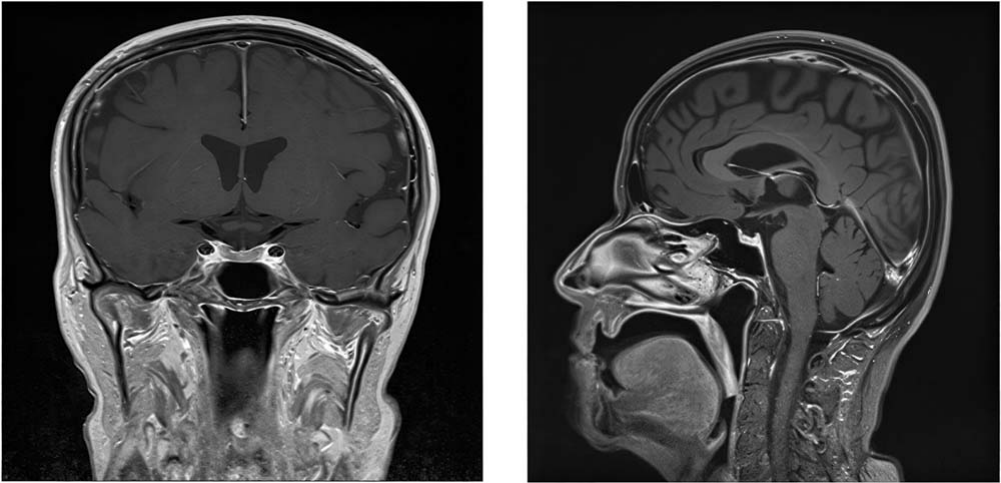
Post-contrast T2-weighted magnetic resonance imaging (MRI) demonstrates normal pituitary.

For diagnostic purposes, we ordered morning ACTH, cortisol, TSH, FT4, electrolytes, LH, FSH, and fasting glucose and, for the exclusion of new lesions, a whole body scan. We assessed the brain using an MRI with contrast. The results showed central adrenal insufficiency, hypothyroidism, and hypogonadism (Table 1). MRI revealed no metastasis or pituitary disorder (Fig. 1).

**Table 1:** Pre-treatment and Post-treatment laboratory results.

Laboratory assays	Prior to Nivolumab	After 6 doses of Nivolumab	Reference range
TSH	1.61	0.12	0.38–4.3 mIU/ml
FT4	15.82	6	12–22 pmol/l
ACTH	-	2.18	7.2–63.3 pg/ml
CORTISOL	271.4	13.63	133–537 nmol/l
Na^+^	139	129.1	136–146 mmol/l
K^+^	4.2	3.92	3.6–5.0 mmol/l
LH	-	4.8	7.7–59 mIU/ml
FSH	-	23.5	25.8–134.8 mIU/ml

ACTH, adrenocorticotropic hormone; TSH, thyroid-stimulating hormone; FT4, free thyroxine; FSH, follicle-stimulating hormone; LH, luteinizing hormone;

The patient has been prescribed hydrocortisone. The total daily dosage was 25 mg, administered in two separate doses: Morning-15 mg; Afternoon- 10 mg. She initiated L-thyroxine at a dosage of 50 mcg one week subsequent to the commencement of hydrocortisone therapy.

Following a two-month resumption of nivolumab treatment post-hormonal stabilisation, restaging at three months revealed that the malignancy remained stable, with no new metastatic sites or progression evident on imaging.

Despite the pituitary biopsy and ACTH stimulation test not being performed, the most logical diagnosis seemed to be immune-related hypophysitis- the temporal relationship to nivolumab exposure, the presence of multiple central pituitary hormone deficits, a normal pituitary MRI, and the absence of metastatic disease.

## Discussion

The incidence of immune-related hypophysitis differs according to the type of ICI. With the CTLA-4 antibody, ipilimumab, the occurrence is high at about 4%–17%; however, with a PD-1 antibody like nivolumab, it is rare at less than 1% [3]. Additionally, typical autoimmune lymphocytic hypophysitis is more common in women, while hypophysitis related to immune checkpoint inhibitors is more common in men, with a ratio of 1.7:1. This trend may be attributed to the fact that melanoma and lung cancer, the tumour categories where ICIs have been used most frequently, are more prevalent in males [4].

Although we don’t fully understand how ICI-induced hypophysitis works, recent research (GTEx—Genetype Tissue Expression—Projects) has shown that CTLA-4 and PD-1, which were thought to only be found in T cells, are also present in other organs like the atrium, pituitary, thyroid, and testis. These findings can partially explain the mechanism of this adverse event [5].

MR Imaging is the best technique to assess patients with suspected hypophysitis, helping to exclude other pathological conditions, such as neoplastic lesions and adenomas. Typical findings suggest hyperintensity on T1-weighted sequences, diffuse symmetric enlargement, and a thickened pituitary stalk above 4 mm, often with a V shape [6].

Imaging alterations in the pituitary may occur before clinical and biochemical indicators of pituitary inflammation appear [7]. However, approximately 30% of patients exhibit a normal MRI, which may not fully rule out the diagnosis of hypophysitis.

It is essential to monitor and report any endocrine adverse events that occur during immune checkpoint inhibitors since the number of patients receiving treatment with ICIs continues to increase. Endocrinopathies can be life-threatening, necessitating that treating doctors recognise their clinical symptoms, diagnose them, and manage them to ensure effective treatment and enhance the outcomes of immune checkpoint inhibitor-induced endocrine adverse effects.
